# Supplementary material for: Patterns of Intron Gain and Loss in Fungi
Source: PLoS Biol. 2004 Nov 30;2(12):e422. doi: 10.1371/journal.pbio.0020422 (PMC532390; doi:10.1371/journal.pbio.0020422)
Supplement: Table S1 — Also available at http://genes.mit.edu/NielsenEtAl/. (4.3 MB ZIP). [file pbio.0020422.st001.zip › NielsenEtAl/html/917.html]

AN0120.1.NCU08268.1.MG01540.1.FG09800.1


```
 CLUSTAL W (1.82) Multiple Sequence Alignments - Introns Inserted


Sequence 1: MG01540.1	375 aa
Sequence 2: FG09800.1	349 aa
Sequence 3: NCU08268.1	406 aa
Sequence 4: AN0120.1	347 aa
Alignment Length: 413 aa
Number Identitical Residues: 102 aa
Alignment Score (without introns) 5822


MG01540.1 	MEQDQTQDQQPQSLADCYREAEVSRLALEGSFEAQSPEKRDALSTAIAAYRACLARISAA
NCU08268.1	-----MSDQEPRSLKATFAAAEEKRLSLENSPPS-PADYADTVSAAIKLYQSCLSQISLL
FG09800.1 	-----MSSEEPQSLRSLYEAAEEKRRALENAFEATSPAYRSDLEAALSLYNSARDRISGL
AN0120.1  	-------MDEPQTLRTVFTDAKRQKTALEARPDTNSDTYRSDVNAAIARFEECQRLVSAL
          	        ::*::*   :  *: .: :**    : .    . :.:*:  :. .   :*  

MG01540.1 	SVFSPNETLEDVRTDYLP~FLLTEHHLAGLLQKQ--PTTGPRARAAVLDESRACYERFLH
NCU08268.1	SLFSSNEGLEDLNTNDLP~YLLVNYHLAELIQKIPSPTPSPAERKHILAQAREAYERYLH
FG09800.1 	ALFSPNEGIEDVSTTDIP~YLLLDAHIAELVQKT--PNQSPDQRLEVLAKSRAAYERFLA
AN0120.1  	SMFSSNELLEDISTGDLP2YLTIEYHIAELLQKS-----YSSDRESTLQRALGQYEKFLT
          	::**.** :**: *  :* :*  : *:* *:**       .  *   * .:   **::* 

MG01540.1 	LLDGYE-ALGQSQARLLHEYDGAPRMFSTLPS--DPARRRDTKIAAFRREKDLRAKLE~A
NCU08268.1	LLDSYEGLLNPTYKKLLERYTDSPTDFSVVSSASDPNARRNAKIANFKAEKELRQKLA~Y
FG09800.1 	AVDGYG-LVQAPYDRVLERYRDEPESFAVVATT-DAAARRDGKIANYRAEKALKEKLE~M
AN0120.1  	RLEDYE-LLSQKDKKLYERYVANPSSFSLTQTN-DAATRRDVKVTRFREEKELKQKLE0K
          	 ::.*   :     :: ..*   *  *:   :  *.  **: *:: :: ** *: **   

MG01540.1 	LKSRSRR----ARQGEDE---------GGDGDDVAEEYARPVYMTQLEMAADEALQALES
NCU08268.1	LRQRPEYAAADLELDDDESISQSRGGGGGGGSTGDEELVRSVHLAHLDLCTHLTFQALES
FG09800.1 	LRRNPRY----LEQGD-------------------EELVRELYMTQITFAVHSALQALDS
AN0120.1  	KLQSDDDD------------------------------LRQLYLAEIKLYTHQTFQSLDM
          	                                       * ::::.: : .. ::*:*: 

MG01540.1 	INREEEVLAQAPEPLMSTGTTVE-----------EDERRRR---AQEDADGASRLDRPLR
NCU08268.1	LNRELEVLNMAPREPASLPSAQLRNNQAGNDGGAQDDLRRRQGGGAADRDYSDRLDMPFS
FG09800.1 	LNREVDILAQAPRPLAPSSTDTP-----------SKD------------DHSYRLDQPLR
AN0120.1  	LAQELSMLSMMKKMPPKPEPSHE-----------VDARGRAK---SNDENYSERLDPPIS
          	: :* .:*    .      .               .     .     . : : *** *: 

MG01540.1 	R-LQSAMGG--PLLNKAGKPLQPFTIVGSRDELAKGVFRPGHNLPTMSIDEYLEEERRRG
NCU08268.1	R-LQGILGGSGPILSKEGRPLRPFTLTGTRQEIAKGVFRPGHNLPTMSIDEYLEEERRRG
FG09800.1 	R-LHSLPGG--PLLSKQGKPLQPFTLVGSRDQMSRDVFRSGHNLPTMSIDEYLEEERRQG
AN0120.1  	QLLKGGKNG--PILSKDGKPLQPFTLLDRRTQLRDGVFRSGHNLPTMTIEEYLAEEHRQG
          	: *:.  .*  *:*.* *:**:***: . * ::  .***.*******:*:*** **:*:*

MG01540.1 	GIIEGGGEASGRIPEP---DEDDEEKADAATYKARQWDEFTEANPRGSGNTLNRG
NCU08268.1	GIIEGGGEASWKAGEKDPNDEDDYEKLDAETMKARAWDEFVEANPKGSGNTLNRG
FG09800.1 	NILQGGVEEKTVI------DEDDYDAVDRETYKQREWDEFVDHNPKGAGNTLNRG
AN0120.1  	NVLKGGKPEPVEI------DEDDMDKADEETMKARAWDEFKEANPRGSGNTLNRG
          	.:::**             **** :  *  * * * **** : **:*:*******
```
